# A Novel Non-invasive Predictor Measure of Left Atrial Pressure and Practical Implicates in High Frequency Ventilation: Theory

**DOI:** 10.7759/cureus.63998

**Published:** 2024-07-06

**Authors:** Gamal Salem, Karim G Salem

**Affiliations:** 1 Pulmonology, Ain Shams University, Cairo, EGY; 2 Research, Microsoft Engineering, Seattle, USA

**Keywords:** stiff index, cental venous pressure wave, high frequency ventilation, pulmonary artery pressure, cardiac output

## Abstract

A new prototype of ventilator mode, based on high-frequency ventilation (HFV), aims to address the interrelations among diverse respiratory variables, several intercardiovascular parameters rapports, and instantaneously the intrarespiratory and cardiac interactions. This article proposes a logarithmic network of integrated correlation ratio H/V, R _correlation_, that primarily relates both dependent respiratory (V) and independent hemodynamic variables (H). Given a constant steady state of the heart and vasculature, a difference between calculated ratio (R calc), and optimized ratio (R opt) could be utilized to adjust many aspects of several parameters and outcomes in critically ill patients. The central venous pressure (CVP) wave analysis aims to approach several cardiovascular parameters, endpoints of left atrial (LA) pressure, and the cardiac output (CO) with the help of thermodilution curve analysis. This might result in several innovative formulas of mathematical base. Their relevant clinical implicates demonstrated a wide area of normalized distribution capacity (Cd), systemic vascular capacity (Cs), pulmonary vascular capacitance (Cp), pulmonary vascular resistance (PVR), and mean pulmonary artery pressure (MPAP). The authors claim that prepared software programming helps network integration of all these variables, feeding back and addressing the prototype of novel mode for the old new application of HFV. One potential unique criterion is adjusting LA and ultimately CO through a feedback control mechanism. Though this all sounds very plausible, the reader must be reminded that everything in this work is based on the most expected speculations and experience and could be potentially different than that in vivo. More importantly, animal experimental studies are warranted.

## Introduction

Although understanding the interaction between the heart and lungs can be complicated, it is essential for clinicians and healthcare professionals. The article is not a clinical trial despite some issues being investigated on populations in some of our studies. It is just a small thought piece aimed at scrutinizing a new concept in high-frequency ventilation (HFV) with a sprinkling of views on cardiovascular and respiratory interplay. The need to minimize human error in the ventilation context is warranted. Circulatory filling pressure is still an abstract quantity in the world of mathematics [1] and does not exist as a physiological driving pressure. The current study aimed firstly to estimate the left atrial pressure (LA) as a primary endpoint, and secondly to ensure the validity of LA pressure in the context of the relationship ratios of hemodynamic to thoracic pressures with their impact on cardiac output (CO).

## Materials and methods

During inspiration with positive pressure ventilation the intrathoracic pressure changes causing changes in venous return (VR), right ventricular output, and pulmonary blood flow. Paradoxically, there may be a reduction in right ventricular impedance with fluid administration, which improves the venous return and cardiac output. 

The speculative inputs in the current article include the demographics of virtual patients, such as age, sex, weight, height, and temperature. The physician selects bias flow (BF), size of the endotracheal tube (ETT), radiological reduction factor RRF% which is the physician's evaluation of radiological lung volume loss, and the frequency(F) in Hz (set with a range of maximum and minimum). The inspiratory/expiratory ratio (I/E), peak inspiratory pressure (PIP) at maximum, the selected R _correlation_ ratio (the ratio of mean CVP to mean Arway pressure calculated in fraction), stiff index, and the mean central venous pressure (CVP in cmH2O) are determined and demonstrated. Dynamic lung compliance (C_lung_ in L/cmH2O) from the preceding volume-controlled mode of ventilation should be recognized.

Given a constant steady state of the heart and vasculature, the levels of mean airway pressure (AWP) and the hemodynamic pressures would approach the same equilibrium values in a series of operational steps. Like domino play, each step would follow several processes controlled by the software circuit of the feedback loop with no repetition of data. Replicability and reproducibility can be tested through the prompt application of the submitted software in this work to the best of our ability.

The following scenarios were created on virtual data based on the most popular findings and personal experience. None of the entered data are real, and the outcomes are mathematic speculation.

Scenario 1: When central venous pressure ( mean CVP) is negative or lower than 5 cmH2O with hypovolemia, it is recommended to use negative R_correlatiion _stiff index 0.5, low bias flow 0.01 ml/s, F =5 Hz, and inspiratory to expiratory ratio (I/E) ratio of 0.33.

Scenario 2: When the mean CVP is higher than or equal to 8 cmH2O, it is recommended to use the I/E of 1:1, higher stiff index, higher R _correlation_, and low bias flow.

Scenario 3: When the mean CVP is measured between 5-8 cmH2O and hemodynamically stable, it is recommended to use an intermediate R _correlation_, I/E ratio of 0.33, and low bias flow.

Scenario 4: When the patient is hemodynamically shocked, the physician increases cardiac output by 15%, and it is recommended to use a lowered R _correlation_, I/E ratio of 0.66, higher stiff index, and an increase of bias flow.

Table 1 demonstrates the input data of the above-mentioned different clinical scenarios with special emphasis on the R _correlation_ ratio and I/E ratio. The R_ correlation _should be registered as negative if CVP is negative, but must be higher selected when mean airway pressure (mean AWP) is expected to be high. The stiff index is selected at 0.5 as default and the higher the lung compliance (C_L_) the lower the stiff index. Bias flow (BF in ml/s) controls and indicates the rate of continuous flow of humidified blended gas through the circuit. Bias flow is selected between 0.01-0.09 ml/s and proportionally increased with increased volume and reduced dead-space-to-tidal-volume ratio (DV%). Note the complex interplay of stiff factor and bias flow is of concern in case of inadvertent high lung volume. The upper and lower oscillation volume (Vosc max ml/kg/cycle) should be determined for the alarm system, and similarly, the same upper and lower frequency limits (Hz) are crucial (Table 1). The size of the endotracheal tube (ETT) is essentially recorded as it constitutes a component of the total resistances, with a range from 2-20 cm/L/s. The temperature should also be reported as the thermal measurements change over time in flowing blood through the thermodilution study.

**Table 1:** Demonstrated respiratory parameters including the demography of virtual patients and the set variables RRF is the radiological reduction factor %, R _correlation _is the ratio of central venous pressure to airway pressure, C_L_ is Lung Compliance, F(Hz) is frequency, I/E is the inspiratory/Expiratory Ratio, PIP max: Peak Inspiratory Pressure at Maximum, R _correlation _is a surrogate ratio of the relation between central venous pressure (CVP) and airway pressure), and ETT is endotracheal tube size. For verification/validation of data please follow this link. Download the file, enter the input data, and press the 'Calculate' button to view the output: https://1drv.ms/u/c/7a1a0756cc016cb9/EVeE9M2Xu9pIpZBKBuatZa4BYpGhhOKuAiRUdMoFPzBozw?e=OroHwZ

Variables	Scenario 1	Scenario 2	Scenario 3	Scenario 4
Gender	Male	Male	Female	Male
Age Y	25	35	78	25
Weight Kg	75	80	72	72
Height cm	167	170	162	162
RRF%	60	35	60	35
Stiff index (fraction percent)	0.50	0.6	0.51	0.65
R (fraction percent)	-0.55	0.66	0.62	0.55
Mean CVP cmH2o	-3	15	7	5
C_L_ Lit/cmH20	0.03	0.02	0.01	0.03
Bias flow ml/s	0.01	0.01	0.01	0.09
Frequency Hz	5	5	5	5
I/E Ratio	0.33	0.5	0.33	0.66
Temperature in Celsius	37	39	36	37
Max PIP cmH20	40	45	40	30
Upper frequency limit Hz	10	10	10	10
Lower volume limit ml/kg	0.8	0.8	0.8	0.8
Upper volume Limit ml/kg	5	5	5	5
ETT size mm	7.5	8	8	7.5

However, justifying parameter selection is a skill of clinical physicians and is outside the scope of the current work and must be left to physician decision, although minimal demographic data are needed to minimize human errors.

Monitoring of CVP waves includes: mean CVP (cmH2O), analysis of the corresponding waves "a" and "v" in mmHg, and the delta CVP, calculated as the difference between the two waves in mmHg. The vital signs including heart rate and systolic and diastolic blood pressure are demonstrated in Table 2. The patients were connected to capnography for measuring end-tidal CO2, and mass spectrometry to measure CO2 (i) % (the inspiratory concentration fraction of CO2), and CO2 (e) % (the expiratory concentration fraction of CO2). The partial arterial pressure of oxygen PaO2, carbon dioxide PCO2, and the inspired oxygen fraction are depicted in ABG gases analysis. 

Table 2 illustrates the data recorded on connecting to capnography and mass spectrometry in the four different clinical settings.

**Table 2:** Illustration of data extracted by ICU monitoring These include the delta CVP or difference between "a" and "v" waves, the heart rate HR, the fraction of inspired oxygen (FiO2), partial pressure of carbon dioxide in arterial blood (PaCO2), and partial pressure of end-tidal carbon dioxide (PECO2) in mmHg measured by capnography. The analysis by mass spectrometer includes CO2 (e)%: the expiratory concentration fraction of carbon dioxide, and CO2 (I)%: the inspiratory concentration fraction of carbon dioxide, the fraction of inspired oxygen (FiO2), partial pressure of carbon dioxide in arterial blood (PaCO2), and partial pressure of end-tidal carbon dioxide (PECO2) in mmHg measured by capnography, systolic and diastolic blood pressure, and record of 'a' and 'v' waves of CVP. For verification/validation of data please follow the link: https://1drv.ms/u/c/7a1a0756cc016cb9/EVeE9M2Xu9pIpZBKBuatZa4BYpGhhOKuAiRUdMoFPzBozw?e=OroHwZ. Download the file, enter the data, accept, and press the button calculate to get the outcome results.

Variables	Scenario 1	Scenario 2	Scenario 3	Scenario 4
Delta CVP mmHg	3.3	4	7	2.3
Heart rate beat/min	120	95	95	120
CO2(i) %	0.8	0.7	0.7	0.8
CO2(e) %	10	17	17	11
PaCO2 mmHg	60	60	50	55
PECO2 mmHg	53	57	43	45
PaO2 mmHg	50	60	62	60
FiO2 mmHg	1	0.8	0.8	1
Diastolic BP mmHg	65	100	95	60
Systolic BP mmHg	85	140	105	75
A wave mmHg	-4	17	9	6
V wave mmHg	-7.7	13	2	3.7

The cardiac output (CO) and volumetric parameters could be measured with the thermodilution technique and be obtained after injection of 50 mL of cold isotonic saline 0.9% (temperature <8°C) via a central venous catheter (Table 3). It causes a change in the temperature of the flowing blood. The data of venous return measurements through a thermistor on the tip of the central venous line cannula inserted in the right ventricle provides a thermos-dilution curve according to the Stewart-Hamilton algorithm. The mean of three consecutive injections is recorded. Volumetric parameters are calculated from the mean transit time (MTt) and the exponential downslope time (DSt) of the thermodilution curve, where T_1 _is the starting time of injection and T_2_ is the time of detection (Table 3).

**Table 3:** Data of thermodilution curve monitoring MTt: the mean transit time of the thermodilution curve; DSt: the exponential downslope time, T1 thermo (second) as 0.5 and T2 thermo (second) as 2.5 by default. T_1_ is the time(s) of the initial thermal distribution test and T_2 _ is the time over the set time or period defined by default, Ci: the temperature at the site of injection; Cn: the temperature at 'n' beat number that diluted the cold injectate with blood at body temperature, and n is the exponential of the number of heartbeat legend to ejection fraction as EF % = (1-EF)^n^. For verification/validation of data please follow the link:https://1drv.ms/u/c/7a1a0756cc016cb9/EVeE9M2Xu9pIpZBKBuatZa4BYpGhhOKuAiRUdMoFPzBozw?e=OroHwZ. Download the file, enter the corresponding data, accept, and then press the button calculate to get the outcome results.

Variables	Scenario 1	Scenario 2	Scenario 3	Scenario 4
Initial blood temperature Celsius	37	39	36	37
Initial injectate temperature Celsius	8	8	8	8
Injectate volume ml	50	50	50	50
T1 thermo second	0.5	0.5	0.5	0.5
T2 thermo second	2.5	2.5	2.5	2.5
Average temperature Celsius	30	32	25	30
Ci Celsius	11	12	12	10
Cn Celsius	23	29	25	25
DSt second	0.55	0.6	0.97	0.42
MTt second	0.7	1.9	1.6	1.1

The symbol ‘’n’’ is the sequence number of the heartbeat following the first heartbeat. It coincides with thermal concentration at the right ventricle Cn(Rv) at the time interval of DSt. Technically, this is to be ensured via the synchronization of the continuous heartbeat recordings, in parallel with the thermal concentration measurements through the thermos-dilution curve analysis. Consideration of measurements should emphasize the sensitivity (delayed time detection) measurement done since the time onset of injectate.** **The C_n_ is the temperature at the “n” beat (in number) that diluted the cold injectate with the blood at body temperature and C_i_ is the initial temperature measured at the right ventricle. They represent the temperatures at the site of injection (RVEDV) and at the site of detection, the end-diastolic right ventricle volume RVEDV_n_ mm/m^2^, respectively. The volume difference between RVEDV and RVEDV_n_ is called delta RVEDV and is expressed in mm/m^2^. ^ ^

As with all advanced hemodynamic monitoring systems, efficacy in improving the patient's outcomes has yet to be conclusively demonstrated, and the metabolic profile should be enrolled (Table 4). The oxygen consumption in ml/min^^-1, ^arterial oxygen saturation (SpO2), mixed venous saturation (SvO2), and hemoglobin in gm/deciliter are reported to estimate the cardiac output. Estimation of fraction of rebreathing CO2 on inspiration (CO2b(i)%) based on end-tidal CO2 on inspiration, and estimation of the fraction of rebreathing CO2 on expiration (CO2b(e)%) on expiration based on partial pressure of end-tidal carbon dioxide (PECO2) during rebreathing is utilized for measuring the cardiac output. This method is somewhat, more suited to sedated ventilated patients, making cardiac output easily measurable. 

The following data illustrates the vital, hemodynamic profile and the blood gases (Table 4).

**Table 4:** Illustration of the vital data and hematological and blood gases It included oxygen consumption ml/min^-1, arterial (SPO2 %) and venous oxygen saturations (SVO2 %), monitored fraction of rebreathing (CO2b(i) %) on inspiration and expiration (e), urine volume per hour (UV) ml, and perspiration Volume (PV) in ml. Cardiac scale % is an option set by the physician to promote increased cardiac output (CO). For verification/validation of data please ensure all the input data from Tables (1) to (4) in the following link, accept, and then press calculate to get the outcomes in the following result section: https://1drv.ms/u/c/7a1a0756cc016cb9/EVeE9M2Xu9pIpZBKBuatZa4BYpGhhOKuAiRUdMoFPzBozw?e=OroHwZ

Variables	Scenario 1	Scenario 2	Scenario 3	Scenario 4
Oxygen Consumption ml/min^-1	80	155	195	130
SPO2 %	0.84	0.86	0.89	0.88
SVO2 %	0.45	0.52	0.55	0.45
Hemoglobin gm/dl	7	11	12	10
CO2b(i) %	0.7	0.4	0.7	0.7
CO2b(e)%	6	6	9	4
UV ml/24h.	15	25	70	5
PV ml/24h.	20	10	25	30
CO Target Scale %	5	5	5	15

## Results

The study included two compartmental issues: respiratory interconnection (Table 1) and intracardiac rapport (Table 2). In this model of ventilation, it was attempted to highlight the present understanding of certain salient aspects of integrated cardiopulmonary physiology, implicating their benefits through a balanced extricable platform of lung and heart in critically ill patients. The output of the present article results includes the estimation of pressure oscillation (P_osc_, cmH2O), PEEP positive end expiration pressure, PEEPi or the intrinsic PEEP, V^.^CO2 (L/min/DCO2^-1) the respiratory equivalent of CO2 production, minimal resistance R_min_ (cmH2O/L/s), frequency (F/Hz), oscillatory volume Vosc (L, ml/kg/cycle), and DCO2, the gas transport coefficient. 

For control of the mean AWP, the peak inspiratory pressure at maximum (PIP _maximum_) is defined by the physician as a set input, and an alarm should work in case the mean AWP is above the upper limit; the lower limit is 5 cmH20 by default.

According to the following equation, the mean AWP can be calculated as:

$mean\, AWP = \kappa (ti\, x\, \frac{(maximum\, PP - PEEP)}{t_{total}}) + PEEP$ [2]** **(Equation 1)

Where t_i_ is inspiratory time, t _total_ is \the entire duration of the stroke volume V_osc_, and kappa Ϗ is the waveform constant that takes a value of 0.5-1.0 in the sinusoidal wave shape.

The Delta amplitude (Delta P vent) is defined as the sum of the maximum inspiratory and maximum expiratory amplitudes and must be double the mean AWP, called peak-peak amplitude:

Delta P vent = 2 × Mean AWP (Equation 2)

The amplitude is the transthoracic pressure or the oscillatory pressure related to the mean AWP and can be estimated from the difference between peak inspiratory pressure (PIP) and mean AWP, sometimes called peak-to-trough amplitude.

Amplitude = PIP - Mean AWP (Equation 3)

The relation of Vosc changes (oscillatory volume) in response to an increase in amplitude is crucial in terms of compliance specific to oscillatory volume. The better the compliance of oscillatory volume the less the pressure amplitude required to achieve the same oscillatory pressure. On the contrary, reduced compliance (i.e., in high frequency) causes a blunt effect on volume changes. This is explained in the following formula [3]:

V_osc_ = C_L calculated_ ΔP (1-e^-ti/RC^) (Equation 4)

Where V_osc_ max ( ml/kg/cycle) is the oscillatory volume, t_i _is the extent of duration in seconds for the inspired volume, the rapidity of volume achieved is called the inspiratory time constant (RC), C_L__calculated_ is the calculated compliance of the lung, and ΔP is the pressure amplitude.

The stiff factor was defined as a proportion of lung tissue elasticity relative to chest wall elasticity to ensure oscillation tidal volume [4]. Accordingly, the equation was formulated as:

Stiff index = 1 - (E _lung_/E_rs_ ) (Equation 5)

Where E _lung_ is lung elasticity and E_rs_ is respiratory system elastance.

Software programming selects the stiff factor “chosen stiff factor” in relevance to the lowest possible dead space volume on one hand, and the highest oscillatory volume on the other hand.

The transthoracic pressure or oscillatory pressure amplitude can be expressed as:

P_osc_ = amplitude × sine 2πft_1/2_ + D (Equation 6)

Where sine is the sinusoidal function of time in radian, f is frequency, π =pi =3.14, and t_1/2_ is half inspiratory time. This corresponds to the highest magnitude of P_osc_ at the middle of the inspiratory or expiratory sine wave, while the vertical shift D is defined as the vertical displacement of the amplitude away from the mean AWP to the mean AWP calculated.

The rate of flow is indicated by a ball float and ranges from 24-70 L per minute (0.4-1.3 L/second) with 5 L per minute (0.08 L/s per second) increments [5]. The current theorem mode suggests utilizing 40-130 L/second (0.04-0.13 ml/second) on the initial setting.

For a change of mean AWP to mean AWP _calculated_, a consequent change of R _min_ must occur in relevance to bias flow according to Equation 7, where bias flow is inversely related to the R _min_. The resistance minimal R_min _is defined as mean AWP divided by bias flow in cmH2O/L/s, and thereafter a consequent change of R_min_ must occur in response to the bias flow selected. 

R _min_ cmH2O/L/s = mean AWP cmH20/bias flow L/s (Equation 7)

Both end-expiratory lung volume (EELV) and functional residual capacity (FRC) times (1- RRF/100) give the corresponding acting or volumes based on radiology, and when multiplied by the correction factor BTPS (the body temperature, ambient pressure, and gas saturated with vapor), it gives EELV _corrected_ and FRC _corrected_.

The sustained inflation lung volume (volume _flex_) induced by PEEP could be calculated as functional residual capacity (FRC _corrected _) minus the residual volume (rV _corrected_)_._

Volume _flex_ = FRC _corrected_ - rV _corrected_ (Equation 8)

The difference between EELV (corrected) and FRC (corrected) precludes estimation of volume _plateau _, which is used to estimate the lung compliance _calculated_._ _

Volume _plateau_ = EELV _corrected_ - FRC _corrected. _ (Equation 9)

**Figure 1:**
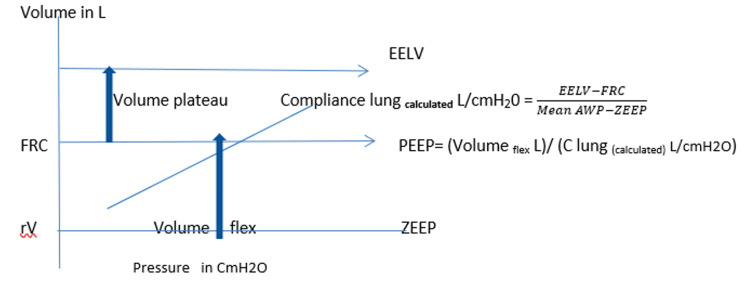
PEEP selection according to presumed EELV corrected and FRC corrected EELV: end-expiratory lung volume; FRC: functional residual volume; ZEEP: zero positive end-expiratory pressure; rV: residual volume; AWP: airway pressure; C lung: compliance lung

We know that static compliance is the ratio of volume change to static pressure change. The volume plateau can be used to calculate lung compliance _calculated._, providing the static pressure gradient between mean AWP and zero positive end-expiratory pressure (ZEEP)** **is detected (Figure 1).

Lung Compliance CL _calculated_ (L/cm H2O) = (EELV-FRC) _corrected_/ (P _plateau_ - PEEP _total_) (Equation 10)

where CL _calculated_ is calculated lung compliance. 

Since FRC is the resting end-expiratory lung volume (EELV) measured at ZEEP( zero end-expiratory pressure), rearrangement will result in:

C lung _calculated_ L/cm H2O = EELV _corrected _- FRC _corrected _/ (Mean AWP - ZEEP) (Equation 11)

Where P (plateau) represents the static continuous distending inflation pressure (mean AWP minus pressure component related to resistance at ZEEP), and where the FRC is the resting end-expiratory lung volume (EELV) measured at ZEEP.

PEEP _total _CmH2O = Volume _flex_ L / C lung _calculated_ L/cmH2O (Equation 12)

An approach in this model highlights the finding that for change of intrinsic PEEP (PEEPi), there** **was a reciprocal relationship with delta change CVP, the difference between "a" and "v" waves. This is important for calculating PEEPi providing a fixed PEEP as per the following equation:

PEEPi _dynamic_ cm H2o = PEEP _total_ cm H2O / ∆ CVP cmH2O (Equation 13)

An automatic adjustment of Fosc (Hz) the oscillatory frequency to the corrected frequency F_corr_ could be done as follows:

Firstly, ti _osc_ can be deduced from equation fitting flow during passive exhalation.

Flow(t) _expiratory_ = (∆P )/t × e- (ti / t ) [2]

Where flow (t) _expiratory_ is the volume represented as flow in the function of time, delta P is the difference between PIP and Mean AWP or the amplitude adjust, ti is inspiratory time, t is the time constant which equals to R max times CL

From the above equation, the inspiratory time ti _osc_ , fitting a flow during passive exhalation, can be calculated as: 

ti _osc_ = ln (( R max⁡ × ∆V adjust insp in L/s )/(amplitude adjust in CmH2O)) × t (Equation 14)

Where Ln is the natural logarithm, R max is the sum of resistances of servo valve on peak inspiration and the total resistance, ∆V adjust insp (ml /s) is flow relative to mean flow (V˙ bias flow), amplitude adjust is the term applied for the sum of delta intrathoracic pressure (ITP) and delta intrapleural pressure (Pit); the two primarily conditioned components of intrathoracic pressure, and t is the time constant and equals R total times C lung. 

Calculation of expiratory time during passive exhalation te _osc _is deduced from ti _osc_ and I/E ratio as follows:

te _osc_ = ti_osc _/ (I/E).

Therefore, knowing ti _osc_ and I/E ratio :

f_osc _= (E/I) / Te _osc_ (Equation 15)

Where te _osc_ is the expiratory time of the oscillatory volume, ti _osc_ is inspiratory time, and E/I is the expiratory-to-inspiratory ratio.

Considering the modification of the alveolar mass equation [6]:

$\frac{V^{\cdot } A}{V^{\cdot } CO2} = \frac{k}{PaCO2(1-(\frac{VD}{VT}))}$ (Equation 16)

Where V^.^A is alveolar minuet volume, V^.^CO2 is the respiratory equivalent of CO2 production and is influenced by frequency F and the square of tidal volume (VT), VD is the dead ventilation volume, and PaCO2 is the partial pressure of carbon dioxide in arterial blood.

Substituting V˙A by (F _corr_ × Vosc _max_ ⁡ ml), this result in:

$Fcorr\, (Hz)\, = \frac{k\,\, x\,\, V'CO2\, (BTPS)}{Vosc\, max\,.\, in\, ml\, x\, (PaCO2(1-\frac{VD}{VT}))}$ (Equation 17)

Since VA/(V^.^CO2)=k/PaCO2(1-VD/VT), and on replacing PaCO2 × (1 - VD/VT)) by PECO2 (partial pressure of end-tidal carbon dioxide), it results in:

$Fcorr\, (Hz)\, = \frac{k\,\, x\,\, V'CO2\, (BTPS)}{Vosc\, max\,.\, in\, ml\, x\, (PECO2) \, in \, Kpa}$ (Equation 18)

Where F_corr_ is the corrected frequency in Hz, K is constant at 863 mmHg, V˙CO2 in L/min, PaCO2 measured in Kilopascal, V_osc_ _max_, the oscillatory volume from the ventilator diaphragm as a function of time expressed in ml. For conversion of mmHg to Kilopascal, divide by 7.501 approximately.

Note that expiratory oscillaton time te _osc_ has to be slower than the initial t_e_, set by F (Hz) and I/E ratio, otherwise, an alarm must operate. The F _corr_ is the applied corrected frequency. This pushes the controller system to change frequency automatically.

Under the condition of oscillatory volume change, the change of frequencies could thus be estimated when the respiratory quotient is fixed under the steady state, and when an increase of V^.^CO2 is associated with increased dead space or alveolar overdistension. However, recognition of increased end-tidal CO2, PECO2 is imperative for increased dead space ventilation but not due to hyperventilation [6].

The DCO2 gas transport coefficient is estimated, wherein the higher the DCO2 the lower PaCO2 and vice versa. It is only linearly related to frequency but increases exponentially with increases in tidal volume. The resting energy expenditure (REE) provides an accurate metabolic classification in mechanically ventilated patients.

Table 5 illustrates the outcome respiratory data of the four different clinical settings.

**Table 5:** Illustrating relevant respiratory parameters outcome. Vosc max: maximum oscillatory volume in ml/kg/cycle; DaLV % peak alveolar distension; Posc(I): pressure oscillation during inspiration; Posc(e): pressure oscillation during expiration;  chosen delta CVP: the best chosen (a-v) waves difference by the spread-sheet of software, C total: total lung compliance (L/cmH2O); PIP osc: peak inspiratory pressure (cmH2O); PIP calculated is the calculated peak inspiratory pressure according to mean airway pressure, R min: calculated minimal resistance; DCO2: gas transport coefficient (ml/kg^2/s); F_corrected_ corrected frequency(HZ), R calc: calculated R ratio; delta VD % rato of dead space to oscillatory volume, REE resitng energy expenditure kcal/day, V^.^CO2: the respiratory equivalent of CO2 production, R opt: optimized R ratio; PEEP: positive end expiration pressure, PEEPi: intrinsic PEEP, and F_osc_: oscillatory frequency for automatic adjustment of F_corr_.

Variables	Scenario 1	Scenario 2	Scenario 3	Scenario 4
Vosc max L, ml/kg/cycle	0.18 L (2.78ml/kg)	0.17 L (2.54ml/kg)	0.14 (2.49 ml/Kg	0.11 L (1.68 ml/kg)
DaLV%	0.77	0.62	0.97	0.55
Posc I (cmH2O)	11.36	41.77	27.41	14.18
Posc e (cmH2O)	2.75	-41.77	3.79	-1.74
Chosen delta CVP (cmH2O)	4.84	5.43	9.51	3.12
C total (L /cmH2O)	0.037	0.014	0.017	0.045
Mean alveolar pressure (cmH2O)	5	22.72	13.74	9.1
PIP osc (cmH2O)	13.19	41.77	31.85	16.18
PIP calculated (cmH2O)	11.44	42.20	38.72	15.35
Mean AWP calculated (cmH2O)	5	22.72	13.72	9.09
R min calculated (cmH2O/L /s)	0.5	2.17	1.37	0.10
Cyclical pressure cost /ventilation unit cmH20/ml	1.12	2.17	0.83	0.83
DCO2 (ml/kg2/s)	8.56	21.15	123.99	27.07
Fcorr (Hz)	4.75	7.96	14.41	9.27
R calc	0.74	0.4	0.44	0.29
Delta VD %	34.73	-11.45	8.25	92.17
REE (kcal/day)	2411	2398.2	1285.72	2319.32
VCO2 (L/min^-1)	-48.2	-84.56	-126.87	-67.19
Modified oxygen index	0.07	0.222	0.13	0.11
R opt	0.27	0.379	0.48	0.48
PEEP (cmH2O)	2.87	11.04	5.5	2.94
PEEP intrinsic (cmH2O)	0.64	2.03	0.57	0.94
Fosc (Hz)	7.04	7.17	9.79	10.53

Intercardiovascular rapport

For the second compartmentalized issue or the intracardiac rapport, reviews of the literature showed correlative changes of central venous pressure (CVP) to those of intrathoracic pressure (ITP) on animals experiment at end-expiration; the regression equation was [7]: 

CVP mmHg = [0.1 ± 0.3 (SD)] + [l.0 ± 0.1 (SD)] × ITP mmHg (Equation 19)

Where CVP is the central venous pressure mmHg, ITP is the pleural pressure change in mmHg, and SD is the standard deviation. On fitting Equation 19 into cmH2O with the exclusion of the negotiable and subtle digits of SD, a multiplication of both sides of the equation by 1.359 (the conversion factor from mmHg to cmH20) gives:

CVP cmH20 = 0.1359 + (1.359 x ITP) (Equation 20)

In extrapolation, the authors propose a salient measurable ratio labeled R _correlation _that relates hemodynamic changes Δ P heam to respiratory pressure Δ vent or (mean AWP changes):

R _correlation_ = Δ P heam cmH2O / Δ vent cmH2O (Equation 21)

Within the mean stream, the interrelationships between CVP and mean airway pressure could be demonstrated as:

R _correlation _= CVP/ mean AWP (Equation 22)

Where R _correlation _could be, in theory, a surrogate of matching perfusion to ventilation ratio. The implication of this formula is determining the mean AWP _calculated_, according to the selected R _correlation_.

Monitoring of right ventricle systolic pressure

Estimation of right ventricular systolic pressure could be performed using the modified Bernoulli equation [8]. The fact is that flow moving across the tricuspid valve must decline by the same magnitude as the transmural pressure gradient. The flow velocity could be utilized for the estimation of right ventricular systolic pressure, whereby blood flow is against a tricuspid valve regurgitation by default. This velocity is estimated by the thermodilution study for the detection of right ventricular end-diastolic volume RVEDV (Table 3). The velocity is estimated using the resistance formula where a linear relation of the length, expressed as RVEDV times DSt and times the fraction ratio of Ci to Cn product, this gives the distance. When this distance is divided by the venous resistance (Rv) mmHg/min/L^-1^, it gives an estimation of the velocity (V) in the tricuspid valve as follows: 

V in mm/m2/s = (( ∆RVEDV (mm )/m2 × DSt (s) × ( Cn thermal)/( Ci thermal ))/ (Rv mmHg/min/L^-1^) (Equation 23)

Where Cn is the temperature after the “n” beat diluted the cold injectate with blood at body temperature and Ci is the initial temperature at the right ventricle.

On applying the modified Bernoulli equation [8]:

PRVS in mmHg= 4 × V^2^ × 10^-2^ + mean CVP mmHg (Equation 24)

Where PRVS is right ventricular systolic pressure.

Estimation of right ventricular diastolic pressure

On the other hand, the value of [mean CVP/ (v+a) / 3] in mmHg [9], was found to be proportionate to tricuspid annular plane systolic excursion (TAPSE).

CVI mmHg = Mean CVP mmHg / ((a+v) mmHg /3) (Equation 25)

Where the CVI is the central venous index representing the signal needed to estimate the ratio of pressure changes to the portal of the right ventricle RV pressure, and the ‘a’ wave measurement should be in its algebraic value whether negative or positive. CVI was found to be correlated with TAPSE with the regression equation [9], as follows:

TAPSE = 1.098 + 0.385 * CVI (Equation 26)

The SVI (Systemic Ventricular Index) represents the noise needed to estimate the ratio of pressure changes to the portal of the right ventricle (RV) pressure.

$SVI \: mmHg = \frac{\frac{1}{7}(\frac{pp}{CVI})^{2}}{CVI\sqrt{2\pi}}$ (Equation 27)

Where PP is systemic pulse pressure, and π is 3.141.

Gamal et al. stated that the mean CVP (mmHg) must be added to or subtracted from SVI to get the right ventricular diastolic index RVDI [9]. 

RVDI = SVI mmHg (+/-) mean CVP mmHg (Equation 28)

The sum versus subtraction is determined when (v) wave is greater than the mean CVP and summation is considered. When the (v) wave is less than the mean CVP subtraction operates in the previous equation.

The right ventricle diastolic pressure in mmHg (PRVD) can be calculated from RVDI as follows:

RVDP mmHg = 1.149 + 0.9932 tmes RVDI [9]. (Equation 29)

The venous return resistance of the systemic circulation Rv in mmHg/L/m^-1^ is calculated as the reciprocal product of right cardiac output divided by the mean CVP as follows:

Rv mmHg/min/L^-1^ = [(mean CVP in CmH2o)/(VR L/min)] / 1.359 (Equation 30)

Table 6 illustrates the cardiac parameters outcome detected by the thermodilution study.

**Table 6:** Cardiovascular outcomes EF%: ejection fraction of right heart; RVEDV: right ventricular end-diastolic volume mm/m2; RVEDV2 right ventricular end-diastolic volume second heartbeat mm/m2; RVESV: right ventricular end-systolic volume mm/m2; n seq: timing(s) of the heartbeats corresponds to the thermal temperature Cn; PRVD: pressure right ventricular diastolic mmHg; PRVS: pressure right ventricular systolic mmHg; RV: venous return resistance of the systemic circulation mmHg/L/min.

Variables	Scenario 1	Scenario 2	Scenario 3	Scenario 4
Right cardiac output (L/min)	2.79	1.94	3.069	2.140
EF % ( rt V.)	0.48	0.60	0.37	0.66
Volume traversed by indicator (ml)	1.59	3.68	4.9	2.35
RVEDV (mm/m2)	15.39	11.65	29.77	8.99
RVEDV2 (mm/m2)	-1.86	-25.51	46.19	-19.94
RVESV (mm/m2)	7.87	4.6	18.46	3.02
Chosen stiff factor	0.5	0.6	0.55	0.65
N seq (s)	1.1	0.95	1.53	0.84
PRVD (mmHg)	-3.15	9.16	4.95	2.97
PRVS (mmHg)	31.7	14.63	20.79	16.17
RV (mmHg/L/min)	0.68	5.68	1.67	1.71

Intra-respiratory and cardiac interactions - calculated R calc ratio

Roger et al. [10] revealed a difference between the CVP measured (CVP _measured_) and CVP calculated (CVP _calc_). The calculated CVP is the measurement corrected for transmitted respiratory pressure change induced by PEEPi according to the following formula.

Calculated CVP mmHg = CVP measure in mmHg - [(PEEPi × CVP)/(plateau - PEEPi) in mmHg)] (Equation 31)

Where (Plateau - PEEPi) is the gradient for inflating driving pressure.

Integration of Equations 20 and 31 results in:

$Calculated\,\, CVP\,\, in\, cmH2O = CVP\, measure\, cmH2O - \frac{PEEPi\, x(0.136+(1.359\, x\, \Delta Pit\, in\, cmH2O))}{(\Delta Pit - PEEPi)\, in\, cmH2O}$ (Equation 32)

Where a value of 0.136 is negligible, and the rearrangement of equation 32 results in:

$Calculated\,\, CVP\,\, in\, cmH2O = CVP\, measure\, cmH2O - \frac{PEEPi\, x((1.359\, x\, \Delta Pit\, in\, cmH2O))}{(\Delta Pit - PEEPi)\, in\, cmH2O}$ (Equation 33)

On the ordinate norms, CVP and ITP are mutually dependent; when one is fixed the other is definitively determined [7]. Having explained the usefulness of the two variables, it is redeemable to direct all efforts to match the airway pulmonary pressure to the hemodynamic changes such that the flow of pulmonary circulation could be maintained at the best of V/Q, the regional matching of the flow of fresh gas to flow of deoxygenated capillary blood. This was the basis of the present work.

Finding a probable governing pattern of mean airway pressure (mean AWP) and CVP changes is an area of interest for charging physicians and researchers. Yet, there is no accurate formula based on the mean central venous pressure for adjustment of the mean AWP in patients under mechanical ventilation.

Therefore, a proposed calculated or R _calculated_ ratio could guide selecting the upper sealing estimate of applied mean AWP as per the following:

On merge of Equations 21 and 33, it gives us:

$R\,calc = \frac{CVP\, measure - \frac{PEEPi\, x\, (1.359 \, x \, ITP)\, in\, cmH2O}{(plateau\, pressure - PEEPi\, in\, cmH2O)}}{mean \, AWP\, cmH2O}$ (Equation 34)

Considering the alveolar mass equation which demonstrates the aberrant changes of alveolar ventilation or the VD/VT ratio [11-12]:

DV/VT = (PaCO2 - PECO2)/ PaCO2 (Equation 35)

Where DV is dead space ventilation and VT is the tidal volume.

Additionally, the relation of the transpulmonary (end-inspiratory pressure) to the dead space volume in artificial ventilation was demonstrated as: 

DV = (7.35 × TPP) + 41.52 [11] (Equation 36)

Where TPP is transpulmonary pressure (the driving pressure for lung inflation).

Integration of Equations 35 and 36 results in:

$TPP = \frac{VT\, in\, ml\, x\, (\frac{PaCO2 - PECO2}{PaCO2}mmHg) - 41.52}{7.35}$ (Equation 37)

Where VT is the tidal oscillatory volume in ml, PaCO_2_ is the partial arterial pressure of CO2, PECO2 is the end-tidal carbon dioxide calculated in mmHg, and TPP is in cmH2O.

Estimation of R optimum

The main purpose of R _calc_ is to guide the level of the mean airway pressure in the complex interplay of linked heart and lung interaction. To render our hypothesis more robust and where airways are opened, the substitution of TPP in Equation 37 with the plateau pressure equation (Equation 33), and replacing the mean AWP by the mean alveolar pressure plus PEEP might result in what we may hypothesize the R _opt_ or Hemo-Vent optimum. This ratio could be remarkably impressive in the interrelationships of hemodynamics and airway pressures upon a more advanced downstream flow, e.g., at the level of pulmonary circulation:

$Ropt (+) = \frac{CVP\: measure - \frac{PEEPi * (0.136 + \Delta Pit\: in\: CmH2O)}{(\frac{Vosc\: max \:in\: ml * (\frac{PaCO2 - PECO2}{PaCO2} - 41.52)}{7.35}-PEEPi)}}{mean\: alveolar + PEEP}$ (Equation 38)

Where V_osc max_ is the maximum oscillatory volume in ml, PEEPi is intrinsic PEEP in cmH20, ITP is intrathoracic pressure in cmH20, PaCO2 is the partial pressure of arterial carbon dioxide in mmHg, and PEC02 is the end-tidal carbon dioxide in exhaled air and is measured by capnography (Table 7)

**Table 7:** Description of several capacities and related pulmonary artery pressures The table demonstrates PVR (pulmonary vascular resistance) in dynes/sec/cm^5^, GEF%: global ejection fraction; SVR: systemic vascular resistance in dynes/sec/cm5; CO(CO2): cardiac output based on Fick's Principle using CO2 (L/min/m^2); CO(O2): cardiac output based on Fick's Principle using O2 (L/min/m^2); Cd: normalized distribution capacity; Cp: pulmonary vascular capacity; Cs: systemic vascular capacity; PAC: pulmonary artery capacity; DPAP: diastolic pulmonary artery pressure; MPAP: mean pulmonary artery pressure; DPG: diastolic pressure gradient (mmHg); PP pulmonary: pulse pressure of pulmonary artery pressures.

Variables	Scenario 1	Scenario 2	Scenario 3	Scenario 4
PVR (dynes/sec/cm^5)	732	171.89	253.7	233.62
GEF %	0.222	0.032	0.066	0.049
SVR (dynes/sec/cm^5)	882	1674.27	1877.2	704.36
CO (CO2) (L/min/m^2)	6.6	4.88	3.97	6.96
CO (O2) (L/min /m^2)	2.15	3.04	3.51	2.22
Cardiac Index (L/min/m2)	0.80	0.51	0.94	0.66
Cd (ml/cmH2O)	0.21	0.003	0.035	0.29
Cp (ml/cmH2O)	0.46	0.026	0.0814	0.24
Cs (ml/cmH2O)	0.31	0.15	0.13	0.42
PAC (ml/mmHg)	1.65	1.90	2.6	1.3
DPAP (mmHg)	17.66	3.92	8.39	2.45
MPAP (mmHg)	22.37	7.5	12.52	7.03
DPG (mmHg)	20.89	0.6	5.60	1.41
PP Pulmonary (mmHg)	14.13	10.71	12.40	13.71

Since the alveolar pressure causes the dynamic-state pressure profiles, the R _opt _has the advantage over R_calc_ as it considers the propensity of lung volume efficiency for any CO2 change to its corresponding CVP _calculated_.

The estimation of Normalized Distribution Compliance Cd(x) is based on recognizing both the systemic vascular capacity Cs and pulmonary vascular capacity Cp. Table 7 illustrates these capacities and pulmonary artery capacity (PAC), diastolic pulmonary artery pressure (DPAP), systolic pulmonary artery pressure (SPAP), and the mean pulmonary artery pressure (MPAP).

Adopting these functional capacitances could provide reasonable explanations for circulatory equilibrium and universality could emerge despite the abstract nature of these pressures and may result in a better understanding of pulmonary circulation. Vascular resistance and compliance of the systemic circulation are seven to eight times larger than those of the pulmonary circulation. We will discuss two different capacitances running in homogeneity, the vascular systemic capacitance and pulmonary vascular capacitance. When compliances are in series, sharing the same flows but different pressure drops, the total compliance is the reciprocal of the two components. In such a series connection the total compliance is less than either component.

The R _calc_ and R _opt_ are resistors of dimensionless unity that surrogate the impedance or resistance of the venous blood flow to match the corresponding airway pressures, since volume is flow in function of time.

Algorithmic approaches for systemic Cs and pulmonary compliances Cp

There are four interrelationships between R _calc_ and R _opt_, demonstrated as follows:

(A) = (R _opt_- R _calc_)/R _calc_ ​​​​: fraction of vascular flow impedance and is consistence with the impedance of mean right ventricular pressure relative to mean CVP.

(Applied when R _opt_ is more than R _calc_). 

(B) = (R _opt_- R _calc_)/R _opt_: fraction vascular flow impedance and is consistent with the impedance of mean pulmonary arterial pressure above right mean ventricular pressure.

(Applied when R _opt _is more than R _calc_). 

(C) = (R _calc_- R _opt_)/R _opt_: fraction vascular flow impedance equivalent and is consistence with impedance of mean right ventricular pressure relative to mean CVP.

(Applied when R _opt_ is less than R_calc_). 

(D) = (R _calc_- R _opt_ )/R _calc_: fraction vascular flow impedance and is consistence with impedance of mean pulmonary artery pressure above right mean ventricular pressure.

(Applied when R _opt_ is less than R _calc_).

These capacitances (A-B-C-D) are defined as flow(volume)relative to the corresponding pressure and they differ when R _calc_ and R _optimum_ are less or more than the other. Note that R _calc _must be fixed to a positive value for a mathematical issue, this contrasts with R _correlation_ which should be set as negative when the mean CVP is negative. These capacitances in terms of (C- D) or (A - B) are running in series in the pulmonary circuit and must be formulated in reciprocal.

In consideration of the above two different situations, the following formulas are postulated for calculating the pulmonary capacity Cp and systemic vascular capacity Cs, as follows:

When R _opt_ is less than R _calc_:

Cp _Less_ ml/cmH20 = (C×D)/(C+D) (Equation 39)

Cs _Less_ ml/cmH20 = Cp _less_ ml/mmHg × RV mmHg/min/L (Equation 40)

Where Cs _less_ is systemic vascular capacitance when R _opt_ is less than R _calc_ in ml/cmH20, and Rv is the venous return resistance of the systemic circulation mmHg/L/m^-1^ and is calculated as the reciprocal product of right cardiac output divided by the mean CVP.

When R opt is higher than R _calc_:

Cp _more_ ml/cmH20 = (A×B)/(A+B) (Equation ​​41)

Where Cp _more_ is pulmonary vascular capacitance when R _opt_ is more than R _calc _in ml/cmH2O.

Cs _more_ ml/cmH20 = Cp _more_ ml/mmHg × RV mmHg/L/min^-1^ ) (Equation 42)

Where Cs _more_ is the systemic vascular capacitance when R _opt_ is more than R _calc_ in ml/cmH20.

Estimation of the normalized distribution compliance Cd(x).

In an extension of CP and Cs, the distribution of the symmetric capacities within the circuit termed normalized distribution capacity is more frequent in practice. Distribution normalized compliance Cd(x) is estimated in ml/cmH2o and is lower than Cs and Cp. The venous return decreases as left atrial pressure and right atrial pressure increase, constituting a venous return plane. The slope of this plan considering the right atrial pressure in Guyton's venous return curves [1] is determined by the reciprocal of the venous return resistance of the systemic circulation (Rv), whereas the slope of the left atrial pressure is determined by the reciprocal of Cs/Cp times RV. The normalized distribution compliance Cd is determined according to whether R opt is less or more than R calc, as follows:

When R _opt _is less than R _calc_:

$Cd\, ml/cmH2O\, (less) = \frac{1}{\frac{CP}{CS}\, in\, ml/cmH2O\, x\, Rv\, mmHg/min/L} \, x10^{-1}$ (Equation 43)

Whereas the slope with consideration of the left atrial pressure, is determined by Cs/Cp times the venous return resistance of the systemic circulation when R _opt_ is less than R _calc_.

When R _opt_ is more than R _calc_:

$Cd\, ml/cmH2O\, (more) = \frac{1}{\frac{CS}{CP}\, in\, ml/cmH2O\, x\, Rv\, mmHg/min/L} \, x10^{-1}$ (Equation 44)

The compliance of pulmonary blood vessels is much smaller than that of systemic blood vessels.

Estimation of pulmonary artery capacity (PAC)

An exponential function is a mathematical function where the input variable $\chi$ occurs as an exponent. It is estimated in the form of f $\chi$ = **a**\^(\chi\)^** **where $\chi$ is variable and “a” is constant called the base of the function and it should be greater than 0.

f ($\chi$) = e^-1/2^ ^[(µ -^$\chi$^)/a]2^ / (a√2π) (Equation 45)

$PAC\: ml/mmHg = \frac{e^{-\frac{1}{2}(\frac{Cp-Cs}{0.142})^{2}}}{0.142\sqrt{2\pi}}$ (Equation 46)

Where PAC ml/mmHg is pulmonary artery capacity, "a" is a constant factor of 0.142, µ is the mean pulmonary vascular capacitance Cp, x$\chi$ is the point of systemic vascular capacitance Cs, π is equivalent to 3.14. Since both vascular compliances of the systemic circulation are seven to eight times larger than those of the pulmonary circulation, the pulmonary capacitance is thus estimated as (1/7-1/8) of systemic capacitance [1], which is equivalent to 0.142 and is thus integrated with the factor “a” constant as 0.142.

Introducing both capacities Cp and Cs for detection of pulmonary artery capacity PAC, where they are connected in parallel, they are thus calculated in subtraction form (Cp - Cs) to give pulmonary artery capacity ml/mmHg (Equation 46). 

Estimation of systolic pulmonary artery pressure (SPAP)

An approach for estimating systolic pulmonary artery pressure is postulated in the following formulas. The blood pressure and blood flow relationship are assumed to follow Ohm’s law. This law means that pressure difference is directly proportional to flow (volume in function of time) and is inversely proportional to resistance.

P = V/R

where V is the volume of blood in the left atrium, LA is left atrial pressure, and R is resistance.

The volume of blood in the left atrium LA (flow in the function of time), stored in the lung in compliance, is assumed to be proportional to its LA pressure. In such context, the indeterminate volume of blood in LA could be estimated as:

$LA\: mmHg = \frac{LA (volume\: indeterminate)\: in\: ml\: /\, Cd \: in\: ml/cmH2O}{Cs\: in\: ml/cmH2O\: x\: PAC\: in\: ml/mmHg} x 10^{-2}$ (Equation 47)

Where Cd is the normalized distribution of compliance, GEF% is the total amount of blood estimated to be present just in prior ventricular systole. It tells us how well the heart is contracting or whether the patient needs inotropes, and is expressed as a percentage. The word “target” signifies the incremental increase of cardiac output that is determined by the treating physician as input data.

If there is a positive mean CVP, the calculation of systolic pulmonary artery pressure SPAP could be approximated as:

$SPAP target = \int_{T1}^{Mtt} [LA\,volume\,indeterminate \,in\,ml\, \, x\, \, Cd\, in\, ml/cmH2O\, target \, \, /\, \, Rv\,\, mmHg^{-1}/min/L \,\,target\,\,x\,\,PP\,pulmonary\,target\,in\,ml/mmHg]\,+(mean\,CVP\,in\,cmH2O\,/\,1.359)]$ (Equation 48)

Where T1 is the initial time of the Thermodilution test, PP pulmonary is the pulse pressure of pulmonary artery pressures, and Cd and Rv, and PP pulmonary data are reported as "target" in the context of renewal cardiac output scale %. 

On the other hand, when CVP is negative, the Rv target will be the nominator while the Cd target would be the denominator in the above equation as demonstrated below.

$SPAP target = \int_{T1}^{Mtt} [\frac{1}{GEF}target \,\,x\,\, CO\,in\,L/min\, \, x\, \, Rv\,\, mmHg^{-1}/min/L \,\,target\,\,x\,\,PP\,pulmonary\, target\,in\,ml/mmHg]\,+(mean\,CVP\,in\,cmH2O\,/\,1.359)]$ (Equation 49) (see later).

Estimation of diastolic pulmonary artery pressure (DPAP)

Systolic pulmonary artery pressure SPAP is usually assumed to be equal to pressure right ventricular systolic (PRVS), when no pulmonary stenosis or right ventricular outflow obstruction exists. Pulmonary artery pulse pressure (PP pulmonary mmHg) could be estimated at the level of PAC (see later). Estimation of diastolic pulmonary artery pressure DPAP is calculated as:

DPAP = PRVS - PP pulmonary (Equation 50)

MPAP = DPAP + (SPAP-DPAP)/3 (Equation 51)

Where MPAP is the mean pulmonary artery pressure mmHg.

Two alternative formulas were used to estimate LA pressure. One is called LA _adjust_ to proofread the concrete LA pressure while the other approach could be estimated from the right cardiac output (see later). This could be applied upon switching to a higher cardiac scale(%), where we can get also the target LA _target _and the target LA _target adjust_

On manipulations of R _correlation_, the physician tends to achieve both the approaches LA and LA_ adjust _closer to LA _target _and LA _target adjust_ respectively. Notice that observed in our model the higher the ∆ CVP the higher the LA pressure, and the lower the differences between LA and LA _adjust_ on hand, and the LA target and LA _adjust target _on the other hand. This might highlight the impact of delta "a-v" wave mmHg for precise estimation of LA pressure. 

The data extracted from the thermodilution technique includes the estimation of the amount of water that is contained in the lungs outside the pulmonary vasculature or** **extra-vascular lung water (EVLW) ml/Kg, using Ideal body weight (IBW,) the lung vascular permeability index PVPI, and calculated unstressed volume fluid USV recommended for I.V. transfusion, indexed to IBW.

Table 8 illustrates increased cardiac output %" and the outcomes or target changes. This is applied on target Cs, PAC, RV, but not Cp. 

**Table 8:** Illustrating left atrial pressures and co-joined relevant pressures within the new deferred cardiac output (target). CO: cardiac output; Cd: distribution capacity of target CO; Cs: systemic vascular capacity; DPAP target: diastolic pulmonary artery pressure on target CO; DPG target: diastolic pressure gradient on target CO; EVLW: extra-vascular lung water; GEDV: global end-diastolic volume indexed to body surface area; GEF%: the total amount of blood estimated to be present just prior to ventricular systole; LA adjust: the alternative estimation of left atrial pressure with thermo-dilution test; LA target: the expected pressure of LA on escalating CO; LA target adjust: the alternative estimation of target LA using thermodilution test; MPAP target: mean pulmonary artery pressure at target CO; PAC: pulmonary artery capacity of target CO; PP: pulmonary pulse pressure of pulmonary artery pressure of target CO; PVPI: pulmonary vascular permeability index; RV target: venous resistance of systemic circulation in target CO; SPAP target: systolic pulmonary artery pressure on target CO; SV target: stroke volume in the target cardiac output; USV: unstressed volume needed to approach the target CO.

Variables	Scenario 1	Scenario 2	Scenario 3	Scenario 4
Rt CO target (L/min)	2.93	2.039	3.22	2..46
Heart rate target (beats/min)	108.9	87	85.9	114
RV Target (mmHg/min.L^-1)	0.65	5.41	3.22	1.49
SV Target (ml)	26.96	23.46	37.5	21.57
Cd Target (ml/cmH2O)	0.23	0.003	0.039	0.223
Cs Target (ml/cmH2O)	0.30	0.14	0.13	0.366
PAC Target (ml/mmHg)	1.47	1.99	2.64	1.95
PP Pulmonary Target (mmHg)	18.33	11.77	14.15	11.05
EF Target % (rt V.)	0.73	0.77	0.53	0.88
GEDV Target (ml)	242.7	1830	1417.8	880
GEF Target %	0.444	0.051	0.105	0.098
DPAP Target (mmHg)	1.27	5.15	4.88	13.38
DPG Target (mmHg)	5	1.72	2.31	12.53
MPAP Target (mmHg)	7.3	9	9.6	17.07
EVLW Target (ml/kg)	-4.82	6.91	6.12	3.35
PVPI Target	-0.77	3.06	1.54	1.38
SPAP Target (mmHg)	15.40	15.86	17.28	27.10
USV Target (ml/kg/hr)	1.1	0.89	2.45	1.2
Left atrium (mmHg)	-3.23	3.32	2.79	1.04
LA Adjust (mmHg)	0.24	2.07	1.29	0.79
LA Target (mmHg)	-3.74	3.42	2.57	0.84
LA Adjust Target (mmHg)	0.15	1.38	0.88	0.35

## Discussion

Further utilization of the R correlation should be tailored according to different clinical settings, particularly in cardiac failure. Ultimately, for a decompensated heart, the mean AWP must be increased while upon improving the heart it should be stepped down. This can be achieved by reducing/ increasing R _correlation_, respectively. This is because it would decrease left ventricular end-diastolic volume (LVEDV) less than left ventricular end-systolic volume (LVES) with the corresponding increase of stroke volume (SV), contrary to the normal heart [13]. This can be processed via a programmed software computer (feedback control system) so that the lower the R calc the higher the mean AWP.

When CVP is readily available, it is assumed to reflect left atrial pressure since left atrial and right atrial pressures are similar apart from certain circumstances, i.e., constrictive pericarditis. The pulmonary arterial time constant which is the product of pulmonary vascular resistance (PVR) and pulmonary compliance (Cp) in the pulmonary circuit (PVR × Cp) is supposed to be constant over a wide range of etiologies and severities of pulmonary hypertension PH [14]; this is because when one rises, the other falls proportionately. Pulmonary artery pulse pressure could be estimated at the level of right ventricular output and is calculated as [15]:

PP pulmonary mmHg = SV in ml/PACml/mmHg (Equation 52)

Where SV is stroke volume, PAC is pulmonary artery capacity, and PP pulmonary is the pulmonary artery pulse pressure (difference between systolic and diastolic pulmonary artery pressures). This is applied regardless of the difference between R _opt _and R _calc_.

Estimation of the left atrium (LA) from VR (right cardiac) output equation

The necessary estimation of LA pressure would not introduce ambiguity in the identification of the other contributing factors or parameters diluting our results, as they are not confounders to divert the conclusion. It was intended to open the circulation loop not only at the right atrial level but also at the left atrial level. If we have no absolute idea what the patient's "normal" left atrial pressure LAP runs at, we should be generous in our estimates. The equation for approximately measuring the left atrial pressure LA, providing no changes of Rv or stressed volume, is estimated from the right cardiac output (VR) as [1]:

$VR \approx \frac{\frac{VR}{4}}{Cs\: ml/cmH2O \: x\: Rv \: mmHg/L/m} - [\frac{mean \: CVP}{Rv\: mmHg/L/m} + \frac{LA}{\frac{Cs}{Cp}\: x Rv\: mmHg/L/m} ]$ (Equation 53)

Where VR in L/m is right cardiac output, Rv is venous return resistance of the systemic circulation mmHg/L/m^-1^, VR/4 in L​ is the stressed volume in L (equates to 0.25% of VR), Cp is the pulmonary vascular compliance, Cs is the systemic vascular compliance, and LA is the left atrial pressure. The right cardiac output VR decreases as mean CVP increases and/or LA increases.

From Equation 53, LA could be depicted according to R _opt_ is either more or less than R _calc_ as:

When R _opt_ is more than R _calc_:

$LA\: _{more}mmHg = [VR\: L/m - \frac{\frac{VR}{4}}{Cs \: ml/cmH2O\: x\: Rv\: mmHg/min/L^{-1}} + \frac{mean CVP\: mmHg}{Rv\: mmHg/min/L^{-1}}] \: x\: (\frac{\frac{Cs\:}{Cp}ml}{cmH2O\: }x \: Rv\: \,mmHg/min/L^{-1}) x 10^{-1}$ (Equation 54)

When R _opt _is less than R _calc_, it is calculated with a reciprocal compliance relationship and the domain becomes as (Cp/Cs), and results in:

$LA\: _{less}mmHg = [VR\: L/m - \frac{\frac{VR}{4}}{Cs \: ml/cmH2O\: x\: Rv\: mmHg/min/L^{-1}} + \frac{mean CVP\: mmHg}{Rv\: mmHg/min/L^{-1}}] \: x\: (\frac{\frac{Cp\:}{Cs}ml}{cmH2O\: }x \: Rv\: \,mmHg/min/L^{-1}) x 10^{-1}$ (Equation 55)

Since VR (right cardiac output) is equivalent to left cardiac output, the estimation of left cardiac output according to the Fick principle by metabolic rate meter could be implemented in the above equation. The base of which is the arterial-venous difference of oxygen content, and is inversely proportional to cardiac output in the absence of shunt [16].

CO (O2) in L/m = (O2 consumption in ml O2/minute)/(∆ (arterial-venous) content per dL× 1.36 × heamoglobine gm/deciliter×10) (Equation 56)

Tedford et al. (2012) [17] stated the presence of elevated left-sided filling pressure shifts the slope of the relationship between right ventricular stroke volume and systolic pulmonary arterial pressure to the left. This results in a less compliant pulmonary circuit (lower pulmonary artery capacitance (PAC) for any given pulmonary vascular resistance (PVR)). This was explored in our software programming which holds an inverse relationship between LA pressure on one hand, and PAC and PVR on the other hand.

For estimating the LA _target_ using the "R" gap (Table 8), following Ohm’s law in turbulent pulmonary vasculature flow, we can get the pressure imposed on the elastance of peripheral pulmonary capillary vasculature or LA pressure. The driving pressure force for pulmonary blood volume, stored in compliance, could be calculated by estimating compatible left atrial blood volume fraction (in distribution to the right side stroke volume) times the CO, which is then divided by fraction functional resistance capacitances as Cd/ (PAC × Cs).

Having an indeterminate LA volume (Equation (47)), a derived equation could be rewritten as:

\(\textit{LA volume indeterminate} = \frac{1}{GEF}\) ** **(Equation 57)

The left atrial pressure _adjust_ was formulated to enhance the validity of LA measurement, since is measured through standard technique or thermodilution test, as follows:

$LA\: adjust = \frac{\frac{1}{GEF} \, \, X\, \, CO\, target\, L/min}{Cs\, \, in\, ml/cmH2O \, x\, PAC \, in\, ml/mmHg} \, x\, 10^{-2}$ (Equation 58)

The LA _target _ , defined as the expected (target) LA pressure on changing CO, could be estimated when considering R _opt_ is more or less than R _cal _, and in the same concept, the LA _target adjust _ with concerns of CO_target_ , the GEF% _target_ , and PAC _target_ formulas could be depicted as follows:​​​

$LA\: target\: adjust = \frac{\frac{1}{GEF} \, \, X\, \, CO\, target\, L/min}{Cs\, \, target \, \, in\, ml/cmH2O \, x\, PAC \, target \, in\, ml/mmHg} \, x\, 10^{-2}$ (Equation 59)

The estimation of pulmonary vascular resistance (PVR) in dynes/sec/cmˆ^5^ as resistance against blood flow from the pulmonary artery to the left atrium could be modeled using the modification of Ohm’s law. The input pressure represents the mean pulmonary artery pressure while the output pressure represents the pulmonary venous pressure which is also equivalent to the pulmonary capillary wedge pressure or left atrial pressure.

PVR in dynes/sec/cm^5^ = (MPAP - LA mmHg)/(VR L/min)] x 80 [18] (Equation 60)

Where total blood flow VR represents the right cardiac output and the digit 80 at the right side of the equation is representative of the conversion factor where one dyne equals 0.0075 mmHg.

**Figure 2:**
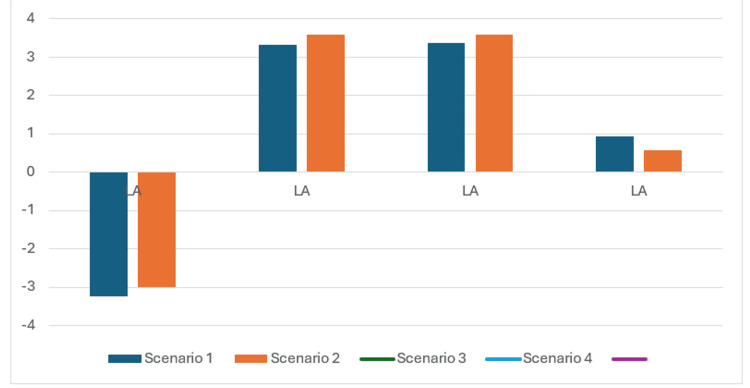
Impact of increased R correlation on LA in each scenario. The left atrium pressure (LA) is increased with Increased R _correlation_, except for shock status where the reverse is true.

Figure 2 demonstrates the scenarios regarding increased R _correlation_ selection Influence on LA.

Closed loop feedback control system of left cardiac output

The changes in cardiac output are best correlated with changes in heart rate [19] and are not correlated to changes in pulmonary artery occlusion pressure. However, change in CO can be mitigated by increasing stroke volume through an increase of unstressed volume resuscitation. In the practice of inotropic drugs inducing change upon target heart rates, it constitutes some limitations in the current mode, particularly in the setting of cardiac compromise, e.g., cardiac decompensation or ischemic heart disease.

**Figure 3:**
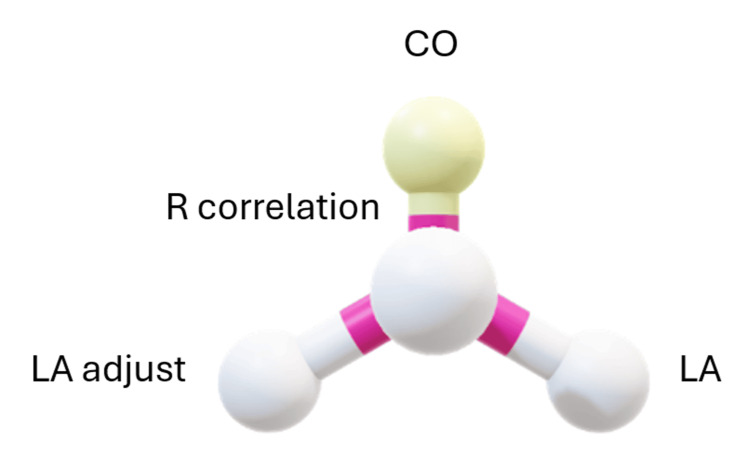
The impact of setting/resetting of R correlation on the LA, and the LA adjust, measured by a different technique with impact on cardiac output CO. The more the R correlation the more the LA and CO, in contrast to shock status. CO is cardiac output measured by Fick's principle, LA is left atrium pressure, and LA adjust is left atrium pressure estimated by thermodilution test.

An alternative approach for enhancing cardiac output rather than a change of HR is monitoring LA in the context of R _correlation_ as input variables. A programmed computer spreadsheet could be applied for resetting the R _correlation_ (Figure 3). Apart from a shocked patient the higher the R _correlation_ the higher the LA and the higher the cardiac output. On the other hand, the decreasing R _correlation_ could lower the LA and the left CO. It is observed in scenario 4 that increasing R _correlation_ would enhance CO through decreasing LA pressure, in contrast to stable hemodynamically patient.

The feedback mechanism could point at narrowing 'the range' of R _correlation. _This enables the treating physician to provide the best chance for approaching most closer value of LA and LA _adjust_, the two independents but validating approaches for LA measurement. This strengthens the validity of the current study. The system looks like a coil that resists stretching with a tendency to keep R _opt_ as lowermost to R _calc_ for the best outcomes.

Narrowing the difference i.e. the more R _optimum_ close to R _calc _the higher the afterload threshold for IV fluid therapy with potential pulmonary vascular edema. The reverse is true provided the slope of the volume pressure relationship of the venous return curve is not changed. A wide difference between both R arms could point to a potential adequate response to IV fluid therapy. 

Clinical implications

One of the applications of "R" window is the estimation of unstressed volume USV and be derived by an equation. The normal response of a healthy individual to fluid resuscitation is to keep right atrial pressure constant as the cardiac output increases owing to increased pressure gradient for venous return to the heart. The difference between recent and prior CO gives the unstressed volume needed to achieve the target CO. The percentage increase of cardiac output, selected by the physician, could help to determine such unstressed volume needed to achieve the target CO. This unstressed volume USV will be added to the stressed volume, equals VR/4, plus the volume perspired PV and volume voided in urine UV in 24 h. (Table 4). Thereafter, the total product will be divided by the ideal body weight IBW to provide the unstressed volume in ml/Kg/hour. This volume is thought to be the net fluid balance in the context of recommended increased or target cardiac output.

 USV in ml/Kg/h = [(CO ml _target_ - VR ml) + (VR ml /4)] + ((UV + PV in ml/h) × 24)) )/(IBW in Kg × 24) (Equation 61)

Where USV is unstressed volume, IBW is ideal body weight, UV is urine volume per hour, and PV is perspiration volume estimated at approximately per hour, providing the stressed volume is constant and the patient is hemodynamically stable with no fluid loss.

We must always consider pulmonary vascular permeability (PVP) and extravascular lung water (EVLW) when diagnosing causes of pulmonary edema for possible detrimental fluid therapy management. PVP can be calculated from the relationship between EVLW and pulmonary blood volume (PBV) derived by the thermodilution technique (Table 8). If the EVLW is elevated without an increase in PVP, the patient has potential cardiogenic pulmonary edema.

To recapitulate, the article explains a reciprocal change of mean AWP in response to setting/ or resetting of the R _correlation_ and helps in frequency resetting in response to expiratory flow and volume changes. It also assists in serving parameters in terms of Cs, Cp, Cd, and PAC.

Limitations

One major limitation might be the cardiac outputs of the right heart and the left heart could be undepicted independently. Without this distinction, it may be difficult to understand the changes in hemodynamics associated with left heart failure or right heart failure. The second limitation is the atrial arrhythmia (if any) that renders desynchrony of the complex interplay of cardiopulmonary integration in this particular work. Thirdly, the work was intended to be performed on HFV, and implementations of these equations on conventional modes of ventilation should be further investigated.

## Conclusions

Respiratory and cardiovascular nous add significantly to one's performance as an intensivist, although debatable. Several implicates could be introduced through the system. The software program backing up this work has been developed to address multiple complexities, helping re-setting parameters, and aiming to select the best input via a feedback loop mechanism. The difference between R _opt_ and R _calc_ is fundamentally utilized to adjust many aspects of essential variables. This ultimately results in stroke volume increase and increased left atrial pressure. This could also be achieved by resetting R _correlation_ or by enhancing LV cardiac output by promoting the scale percentage as input. The closed feedback loop could discover detrimental oscillatory volume, frequency, stiff index, and bias flow through the alarm attention. The key message is broad navigation in the integrated cardiopulmonary parameters that could be ancillary help for learning in the world of relevant physiology.
